# Revisiting effects of teacher characteristics on physiological and psychological stress: a virtual reality study

**DOI:** 10.1038/s41598-023-49508-0

**Published:** 2023-12-14

**Authors:** Lisa Bardach, Yizhen Huang, Eric Richter, Robert M. Klassen, Thilo Kleickmann, Dirk Richter

**Affiliations:** 1 Hector Research Institute of Education Sciences and Psychology, University of Tübingen, Walter-Simon-Straße 12, 72072 Tübingen, Germany; 2 University of Potsdam, Potsdam, Germany; 3 University of York, York, UK; 4 University of Kiel, Kiel, Germany

**Keywords:** Human behaviour, Physiology

## Abstract

Identifying personal characteristics associated with teachers’ stress is a longstanding research goal with important implications for practice. The present work revisits the effects of individual characteristics in terms of neuroticism, classroom management self-efficacy, and cognitive (reasoning) abilities on stress using virtual reality (VR). Relying on a sample of 56 German pre-service teachers (*M*_*age*_ = 22.73*, SD*_*age*_ = 4.93; 50.9% females), we capitalized on a VR classroom environment that allowed the integration of experimental control and authentic teaching situations, where pre-service teachers responded to the disruptive behaviors of the student avatars. We focused on stress responses in terms of psychological stress (self-reported stress) assessed after the VR session, and physiological stress (heart rate) assessed during the VR session. A total of 30 (26) participants was assigned to a condition with higher (lower) levels of disruptive student behavior, referred to as higher and lower complexity condition, respectively. Results from linear mixed-effects models revealed that neuroticism positively predicted psychological and physiological stress responses in pre-service teachers, whereas classroom management self-efficacy and cognitive (reasoning) abilities were not significantly related to stress responses. Level of complexity and the interaction between complexity and individual characteristics did not have an effect. This study underlines the value of VR as a tool for psychological research and contributes to existing knowledge on teacher characteristics and stress.

## Introduction

Teaching can be highly stressful and demanding. Multiple studies conducted in different countries show that between 30 and 60% of teachers report experiencing high levels of stress^1,2^. For example, the 2023 State of the American Teacher Survey found that 58% of teachers indicated that they are exposed to frequent work-related stress^3^. In another study, approximately 30% of teachers reported that the teaching profession is “very stressful” or “extremely stressful” (on a 5-point rating scale, ranging from “not at all stressful” to “extremely stressful”^2^). As compared to other professions, being a teacher has consistently been identified as one of the high-stress professions^2–4^. Prolonged stress is a significant health issue and has been linked to depression, anxiety disorders, and the onset of physical diseases^5,6^. Work-related stress also represents an important factor contributing to teacher attrition^7^, with some estimates suggesting that up to half of new teachers quit their job within the first five years^8,9^. In summary, it is important to find ways to reduce teacher stress as experiencing high levels of stress hinders individual teachers’ thriving at work and negatively affects their quality of life, career-related decisions and professional well-being, and their positive interactions with students. From a socio-economic perspective, it can also be argued that teacher attrition and health problems related to teachers’ work-related stress have immense financial ramifications^10,11^ and negatively affect teaching performance and students’ academic success^12^; hence, decreased levels of teacher stress should lead to reduced societal costs.

Different approaches to counteract teacher stress located at different levels (context, individual) can be distinguished^13,14^. For instance, attempts to reduce teacher stress can center on improving working conditions and limiting the number and severity of stress-inducing aspects of the work. It is also possible to focus on the individual and to help teachers develop personal resources and skills that enable them to better deal with stressful situations. Ideally, approaches to reduce teacher stress combine both the individual and contextual level (and consider their complex and dynamic interplay). However, it can, at times, be difficult or even impossible to initiate major school-level and broader socio-political changes feeding into reformations of the educational system and enhanced working conditions. Therefore, zooming in on individual characteristics relevant to stress seems worthwhile. In addition, certain features relating to the self (e.g., one’s belief systems or motivations) are under individuals’ control to a higher extent than are contextual features, and thus, a focus on individual features can strengthen feelings of personal agency.

Against this background, identifying individual characteristics that make teachers more versus less prone to experiencing high levels of stress becomes an important goal for psychological research with far-reaching implications for practice. For example, research can identify individual teachers who are at higher risk of suffering from stress and can inform the design of interventions for teachers to develop certain characteristics^15,16^. The malleability of motivational and affective (teacher) characteristics has long been acknowledged^17^, but recent years have also seen an increase in interventions that target Big Five personality traits (e.g., conscientiousness, neuroticism, extraversion^18,19^) which have traditionally been considered as more fixed^20^.

The significance of individual characteristics for stress responses has been backed up by several theories and models. The Trait-State-Anxiety theory, for instance, outlines that specific personal characteristics (anxiety as a trait) prompt individuals to react with anxiety and stress in response to varying situations^21^. In a similar vein, recent conceptualizations of the Job-Demands-Resources theory^13^ acknowledge the importance of specific individual characteristics—so-called personal resources—in shaping employees’ workplace experiences, including their stress responses^22,23^^.^ A suggested by the transactional model of stress^24^, stress occurs if individuals perceive that external circumstances and demands exceed their personal coping resources. Thereby, Lazarus and Folkman^24^ emphasize the interaction between the individuals and their personal characteristics and the environment. Further, according to the stress generation hypothesis, a relation between individual characteristics and exposures to stressors exists whereby individuals with certain characteristics are more likely to be exposed to stressful events and to construct events as stressful^16,25,26^. In our study we are interested in effects of individual characteristics on stress, guided by the assumption that certain individual characteristics affect stress responses (i.e., main effects of individual characteristics). However, we also acknowledge the possibility of person x situation interactions in that individual characteristics could interact with situation characteristics (e.g., situations involving more versus fewer stressors).

The present study focused on three teacher characteristics that are conceptually and empirically relevant for teacher stress and that tap into three distinct and complementary domains of individual differences. First, neuroticism (from the Big Five personality framework^27,28^) has been identified as a key risk factor for experiencing elevated stress levels^16^. Neuroticism plays an important role in determining the lens through which stressors are perceived and assigned meaning, and thus, exert effects on individual differences in how people respond to stressors. More specifically, individuals scoring high on neuroticism tend to experience higher levels of stress due to their negative filter, which makes them vulnerable to experiencing stress^16^.

Second, the motivation variable self-efficacy has been conceptualized as an important resource for teachers to deal with stressful situations^23,29^. Self-efficacy captures the degree to which individuals believe they are capable of succeeding in a specific situation or reaching a specific goal^30^. Although teachers are exposed to multiple stressors^31^, disruptive student behavior ranks high among the stress-inducing factors in teachers’ professional lives^32,33^. Therefore, higher levels of self-efficacy for classroom management^34^ should aid in counteracting stress in teachers. Self-efficacy for classroom management, a sub-component of teaching self-efficacy, describes the extent to which a teacher feels capable of dealing with disruptions and misbehaving students, and of maintaining order in class^35^.

Third, cognitive abilities are the most potent predictor of job performance^36^ although research on teachers’ cognitive abilities and teaching effectiveness has been less conclusive^37^. Nonetheless, higher cognitive abilities may enable teachers to better deal with complex teaching tasks^37,38^, which could decrease their stress levels in challenging teaching situations. In the present study, we focused on cognitive abilities in terms of reasoning abilities (assessed using a matrix reasoning task^39^). Whereas classroom management self-efficacy and neuroticism are viable targets for interventions, we included cognitive (reasoning) abilities mainly with the aim of advancing knowledge on cognitive ability correlates in the teacher domain and not because we consider it promising to develop interventions to increase cognitive abilities^40^ or select teachers and pre-service teachers into the profession or teacher education program based on their cognitive abilities.

Most research on teachers characteristics and stress has relied on survey-based studies linking teacher characteristics to their retrospectively reported psychological stress responses^15^. For instance, as demonstrated in a recent meta-analysis, neuroticism displayed a significant positive association with stress, which was most pronounced for self-reported psychological stress responses^16^. Further, an integrative review synthesized findings from existing meta-analyses and systematic reviews on teacher characteristics and concluded that teachers’ self-efficacy seems consistently negatively related to psychological stress^15^. Moreover, laboratory research with samples other than teachers in which stress was experimentally induced has demonstrated effects of selected individual characteristics (e.g., neuroticism) on self-reported psychological stress as well as physiological stress (e.g., heart rate reactivity)^41^. However, even though both lines of research—retrospective survey studies and laboratory studies—have made important contributions to the current state of knowledge on (teacher) characteristics and stress, they are also limited in several regards. On the one hand, survey-based research in naturalistic settings cannot control for potential confounding factors such as differing levels of problematic student behavior in class, varying degrees of support from colleagues, and the dynamic effects of student–teacher interactions^42^. On the other hand, based on the literature on lab studies^41^, we argue that research on stress in the laboratory can be criticized for the reliance on stress tasks with little significance for everyday-life and the challenges that individuals encounter in their profession.

Research using virtual reality (VR) technology provides a remedy. VR, a new track in psychological research, uses digital technology to create complex, realistic, and immersive environments. These environments are under full experimental control, while enabling participants to behave naturally, thus boosting the ecological validity of the results^43^. In educational psychology research, VR provides the perfect balance between experimental control and a teaching and learning situation that can be perceived as much more authentic than what can be achieved in a “regular” experiment^44–46^. Accordingly, VR holds great promise for research on teachers and pre-service teachers and opens up myriad opportunities for situated practice in teacher education^45,47^. However, despite the promise of VR, it should be acknowledged that it is still not a “real” teaching situation. Further, although a VR scenario may aim to confront participants with certain situation characteristics, such as authenticity and complexity, whether the participant does (or does not) subjectively construct the situation as authentic and complex is the results of a subjective interpretation or evaluation of the situation^48^. Importantly for the present study, the usefulness of VR for research on teacher stress has recently been demonstrated in a number of studies. Relying on a VR classroom setting, Huang et al. showed that class size (number of student avatars) influenced psychological and physiological (heart rate) stress responses in pre-service teachers, with higher stress levels in the condition with a larger class size^45^. However, studies exploiting the potential of VR to revisit effects of teacher characteristics on stress are currently lacking.

The purpose of the present study was therefore to examine the extent to which pre-service teachers’ neuroticism, classroom management self-efficacy, and cognitive (reasoning) abilities predict stress responses in a VR teaching session. Stress, a multidimensional phenomenon, manifests itself on the physiological level as well as on the level of subjective experience^49^*;* hence, in our study, we considered both psychological (self-reported stress) and physiological stress responses (heart rate). As students’ disruptive behavior and discipline problems in class are among the most critical stressors for teachers, and especially for new teachers^32^, the set-up of the VR classroom was well-suited to study effects on stress. Roughly half of the sample was assigned to a condition with higher levels of disruptive student behavior and the other half of the sample was assigned to a condition with lower levels of disruptive student behavior (*n*_*low*_ = 26, *n*_*high*_ = 30). These two complexity levels differed only in the number, severity, and concurrences of disruptions, allowing for insights into whether effects of teacher characteristics on stress generalize across situations with more versus less complexity, and to test individual characteristics x condition interactions. Both conditions have been justified in previous studies^44,45^ for subjective perceptions of authenticity and complexity with samples of similar demographics and teaching tasks alike. Aside from the levels of disruptive student behavior, all other contextual factors remained consistent between the two conditions.

Based on theory and prior empirical findings, we hypothesized that neuroticism should make pre-service teachers more vulnerable to experiencing stress. Hence, we hypothesized that neuroticism should positively predict physiological and psychological stress (i.e., positive stress-exacerbating effects)^2^. By contrast, we assumed that classroom management self-efficacy and cognitive (reasoning) abilities should function as personal resources that make individuals less likely to experience stress. Specifically, we hypothesized that classroom management self-efficacy ^23,29^ and cognitive (reasoning) abilities^37,38^ should negatively predict physiological and psychological stress (i.e., negative stress-reducing effects). In addition, we examined effects of situational characteristics (complexity, i.e., higher versus lower levels of disruptive student behavior) on stress and tested whether individual characteristics interact with situation characteristics^24^.

## Methods

### Participants

The current study focuses on pre-service teachers who are enrolled in university-based teacher education programs and have little prior teaching experience. Participants were recruited from a weekly seminar on classroom management held at a public German university. The seminar was a regular course offered every semester and we used data from two cohorts from the same academic year. The seminar forms part of the module on educational theories in the teacher training program; it did not contain a field practicum component and therefore provided no opportunity for real-life teaching (*N* = 56*, M*_*age*_ = 22.73*, SD*_*age*_ = 4.93; 50.9% female, 98.2% Bachelor students, *M*_teaching hour_ = 4.2). These demographic features are representative of this population^44,50^. Due to its accessibility and representation of the target population, this sample was chosen. Furthermore, a focus on pre-service teachers is particularly valuable as research in this population can inform possible preventive measures that could be taken early on because they are yet to start their careers. Although the VR session was presented as an integral part of the seminar, participants could choose freely whether to participate in the VR session and the study with no further incentives. The study was approved by the ethnic review board of the university. Informed consent for study participation was obtained from all participants.

### VR environment and equipment

**Figure 1:**
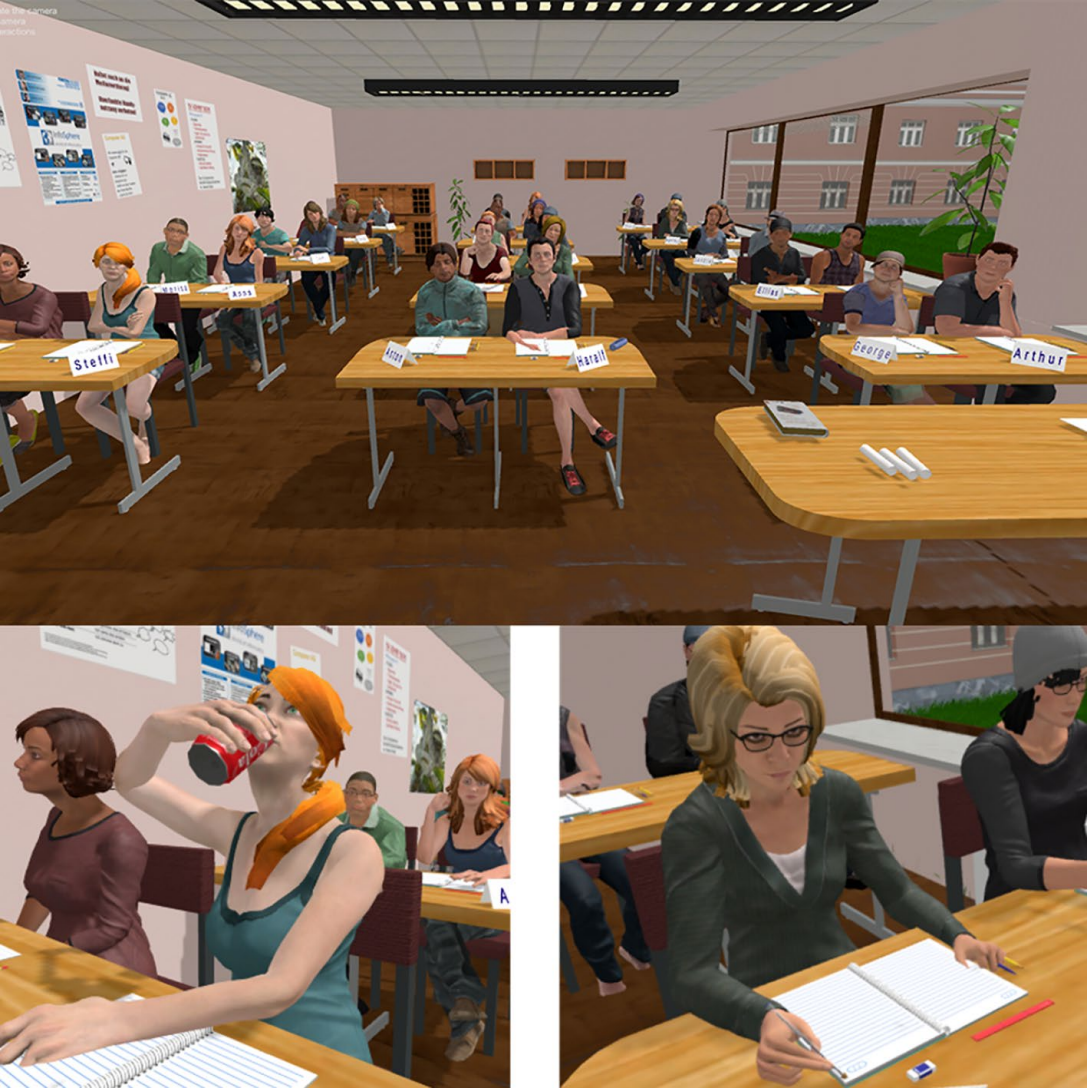
View in the VR Classroom. All views were displayed from the participant’s visual perspective. Top: front view from the teacher’s desk; bottom left: student avatar performs off-task behavior; bottom right: on-task behavior.

Our VR classroom mimics a typical secondary classroom in Germany with a class size of 30 student avatars^51^. Student avatars possess diverse physical characteristics (see Fig. 1), and their behaviors were scripted (see Supplementary Table S1). Pre-service teacher participants (with similar demographic characteristics as our sample) from previous studies^44,45^ indicated that this VR classroom was authentic and convincing. Participants experienced the VR classroom through the HTC VIVE Pro Eye system which allows users to move freely in the real-world environment. The headset has a display resolution of 1440 × 1600 pixels per eye, with a 110° field of view.

### Procedure and measures

**Figure 2:**
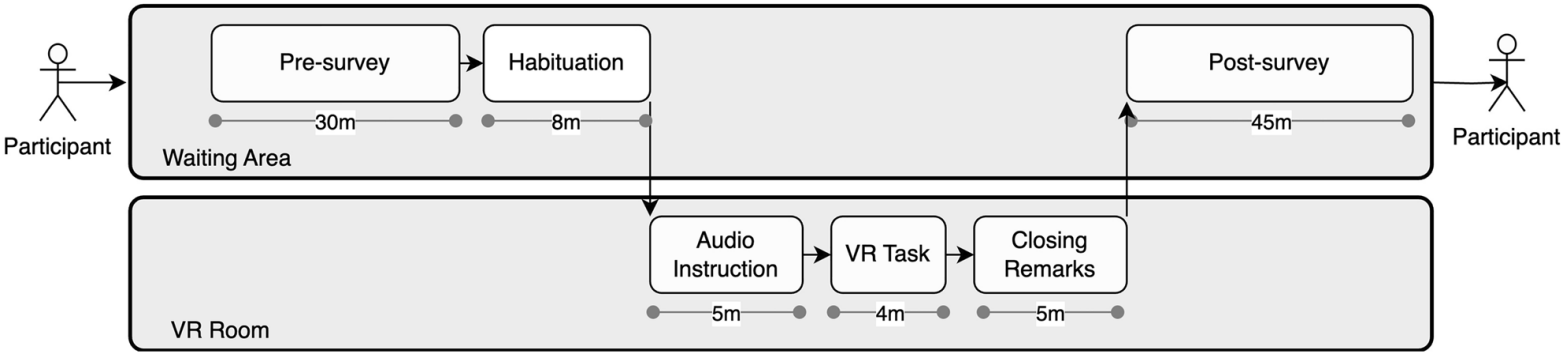
Experiment procedure. Physiological stress (heart rate) was continuously measured from habituation until the end of the VR task.

Participants completed a questionnaire containing measures for classroom management self-efficacy, neuroticism, and worked on a test assessing cognitive (reasoning) abilities prior to the VR session (see Fig. 2). *Classroom management self-efficacy* was assessed using four items from the German Teacher’s Sense of Efficacy Scale^52^ (e.g., “I am confident in my ability to control disruptive behavior in the classroom.”; α = .81), which were rated on a 5-point scale (1 = not convinced at all to 7 = fully convinced). We measured *neuroticism* using the three-item scale from the Big Five Inventory-SOEP^53^ (e.g., “I see myself as somebody who worries a lot.”, α = .69). The items were rated on a 7-point Likert scale ranging from 1 = *s*trongly disagree to 5 = strongly agree. We used the matrix reasoning test from the International Cognitive Ability Resource^39^ (11 items, sum score of correct answers) as a proxy of participants’ *cognitive (reasoning) abilities* (for large validation studies, see ^39,54^). The test contains Matrix stimuli that are similar to those used in Raven’s Progressive Matrices^39,54^. Stimuli are 3 × 3 arrays of geometric shapes, and one of the nine shapes is missing. Participants were instructed that they should identify which of the six geometric shapes that are presented as response choices will best complete the stimuli. Participants also reported their semesters of study (“What semester of your teaching degree program are you in?”) and *VR experience* (“What experience do you have with virtual reality?”, response options: 1 = none, 2 = a bit, 3 = a lot) which were used as random effects in the linear mixed-effects model (see Analyses below for details). Gender was also reported and used as a covariate to control for potential gender differences in stress perceptions. The participants had 30 min time to complete the measures reported above, including the cognitive abilities (reasoning) tests.

During the individual VR teaching session that took place in a quiet room with one experimenter, participants first followed a 5-min audio instruction to familiarize themselves with the VR classroom. They then gave a 4-min lecture about COVID-19 vaccinations during which they needed to respond to typical classroom disruptions. The lecture topic and content were pre-selected and the same for all participants, who were given the presentation slides and lesson plan a week before the experiment. This procedure was designed based on piloting and prior studies^45^. Student avatars would engage in both on- and off-task behaviors (Fig. 1). The disruptions were chosen from a collection of common disruptive behaviors that were evaluated by teachers as being obviously unrelated to the lesson and causing substantial interruptions to the class^55,56^. Avatar behaviors were independent from the teachers’ actions. Participants could react to disruptions by moving closer to students, calling their names, making eye contact, or in any way they deemed suitable.

Participants in the first semester (i.e., the first cohort) were assigned to VR scenarios of lower complexity level while the participants from the second semester (i.e., the second cohort) were assigned to a higher complexity level (*n*_*low*_ = 26, *n*_*high*_ = 30). The high and low complexity level differed in the a) total number of disruptions (30 vs. 15); b) the percentage of significant disruptions, such as hitting and throwing paper balls (10 out of 30 vs. 3 out of 15); and c) the number of occasions when multiple disruptions occurred at the same time (5 vs. 1). Aside from the complexity of the classroom management scenarios, all other aspects remained consistent between two levels. Combining both complexity conditions within one study increased statistical power due to the larger sample and allowed to explore whether effects of cognitive and non-cognitive characteristics on stress generalize across situations with more versus less stressors.

After the VR session, participants rated their immediate psychological stress responses associated with the VR session using four items from Dundee Stress State Questionnaire^57^ (e.g., “I felt tense.”, α = .90). The items were rated on a 7-point scale, ranging from 1 = strongly disagree to 7 = strongly agree. To capture *physiological stress,* we measured the average heart rate (HR) in beats per minute (BPM) during the VR teaching task after controlling for the baseline HR for each participant. Specifically, HR was measured at 0.3 s intervals using Polar OH1—an optical HR sensor worn around the arm that has been validated with electrocardiography^58^. We evaluated each participant's baseline HR to adjust for individual variability in HR induced by variables unrelated to the experimental manipulation^59^. The baseline HR was the average BPM during the last 3 min of an 8-min standardized habituation phase^60^ in which participants were told to sit quietly, avoid moving, and keep their hands on the chair and feet flat on the floor. Finally, the average HR during the VR teaching task was centered on the baseline HR to obtain the measure of physiological stress. HR has already been successfully examined and discussed as a valid measure of stress in various studies^61^.

### Analyses

#### Linear mixed effects models

We relied on linear mixed-effect models to address our hypotheses. In a traditional linear model, the response is modeled as a linear combination of predictors with weights that are referred to as effects or coefficients^62^. In contrast, a linear mixed-effects model (LMM) takes into account both fixed and random effects, in other words, effects that are constant or varying for groups in a population^63,64^. As reviewed by Kliegl and colleagues^65^, LMM is superior to analyses of variance (ANOVAs) because of its advantages such as tolerance of an unbalanced experimental design due to missing data^66^, and researchers’ capability to examine effects of both categorical factors and continuous variables (covariates), as well as their interactions, with a significant gain in statistical power^64,67^. LMM is particularly robust against missing data. In a simulation with 1000 runs of a mixed-effects model, Baayen and colleagues^67^ found that the main effect was almost always detected with and without 20% missing data (missing at random). Its power is only minimally reduced in the situation of missing data. Despite the fact that power is at its maximum, the Type I error rate is within the nominal range. To use LMM, we examined the Q–Q plot of the residuals as well as the plot of residuals plotted against fitted values. We found that the homoscedasticity and normality assumption was met.

In the present study, we treated effects of classroom complexity, neuroticism, classroom management self-efficacy, and cognitive (reasoning) abilities as fixed effects, while semester and VR experience were treated as random effects. Semester and VR experience in this design were “random” in the sense that they were assumed to be a random sample of the semester and VR experience level that might have been included in the study^68^. By modeling random effects along with the fixed effects, we can obtain an estimate of the mean difference in complexity, neuroticism, classroom management self-efficacy, and cognitive (reasoning) abilities; as well as an estimate of the variances surrounding that difference, due to the individual features of each semester or VR experience^69^. Models were fitted with restricted maximum likelihood (REML) using the lmer function from the lme4 package^70^ in R 4.1.2^71^. We included Akaike Information Criterion (AIC; decreases with goodness of fit) and the Bayesian Information Criterion (BIC; decreases with goodness of fit) as goodness of fit indices^71^.

#### Models tested in the current study

We constructed and tested three sets of LMM models for each stress reaction: Model 1 (M1) included only the fixed effects of interested variables; Model 2 (M2) added the control variable of gender; Model 3 (M3) additionally included the interaction term neuroticism/cognitive (reasoning) abilities/self-efficacy × complexity to examine the potential effects of individual characteristics × situational conditions. To clarify whether the introduction of further components into the model would significantly improve model fit, we conducted the likelihood ratio (LR) test to compare the three models.

#### Effect size calculation for fixed-effects terms

The effect size estimate (*d*) that extended Cohen’s *d*^73^ to mixed-effects model was conceived by Judd and colleagues^68^. This estimate might be utilized for designs where the two random factors are crossed and designs where one random factor is nested within the other. Please see Equation 5 in Judd and colleagues’ original paper for the mathematical expression of this definition. An example of the calculation can be seen in Brysbaert and Stevens’s^74^ replication study.

#### Missing data handling

Using Little's^75^ missing completely at random (MCAR) test, we performed missing value analyses to uncover potential patterns in missing data that might influence the analysis. In the case of a non-significant Little’s MCAR test, observed data is considered to be a random sample from all data, allowing for a complete-case analysis with no bias imposed^76^. In the current study, we obtained incomplete HR recordings in 8 observations due to technological failure (14.29%) and these were excluded from the analysis. Same for psychological stress, one participant was excluded from the analysis due to an incomplete record. MCAR test yielded non-significant results for all variables included in our models, therefore the data were considered to be missing completely at random (χ^2^ = 33.27, df = 8, *p* = .21). Additionally, the missing data is not likely to bias the estimation due to the robustness of LMM against missing data^67^.

### Ethical approval statement

This study adhered to all national and international regulations for protecting human subjects. The study was approved by the ethnic review board of the University of Potsdam. Informed consent for study participation was obtained from all participants.

## Results

Descriptive statistics and bivariate correlations of all variables are shown in Table 1. Data were analyzed using linear mixed-effects modeling (LMM) to evaluate the main effect of neuroticism, classroom management self-efficacy, cognitive (reasoning) abilities, and classroom complexity on stress (fixed effects), while accounting for covariate effects that could be generalized to other semesters and VR experiences (random effects; see Methods for details). Three LMM models were tested for physiological and psychological stress responses, respectively: Model 1 (M1) included only the fixed effects of the variables of interest. In Model 2 (M2), we added the control variable of gender. Lastly, Model 3 (M3) additionally contained the interaction terms neuroticism/cognitive (reasoning) abilities/self-efficacy × complexity. Likelihood ratio (LR) tests were conducted to compare the three models and to examine whether the introduction of further components into the model significantly improves model fit. Effect sizes were calculated following standard procedures for mixed-effects models^74^ (see Methods for details). All analysis files and the anonymized data set are available at the Open Science Framework (OSF, https://osf.io/vkbwx/).

**Table 1:** Descriptive statistics of all included variables and bivariate correlations (fields 1–7 =).

Variable name	Range (scale range)/category	*M*(*SD*)	Missing (%)	1	2	3	4	5	6	7
1. Semester	1–21	4.76 (3.68)	0 (0%)		.14	.20	.06	.24	.01	− .08
2. VR experience	1–3 (1–3)	1.31 (0.51)	1 (0.02%)			− .07	.03	.17	− .02	− .10
3. ^1^Neuroticism	1.33–4.67 (1–5)	2.94 (0.78)	0 (0%)				− .38**	.10	.01	.23
4. ^1^Self-efficacy	1.25–6.00 (1–7)	3.87 (1.23)	0 (0%)					.26*	.49***	− .08
5. ^1^Reasoning abilities	1– 10 (1– 10)	5.95 (2.39)	1 (0.02%)						.35**	.13
6. ^1^Psychological stress	1.50–6.50 (1–7)	3.49 (1.29)	0 (0%)							.10
7. ^1,2^Physiological stress	− 10.72–62.40	16.90 (14.23)	8 (14.29%)							

*N* = 56. ^2^Variables displayed in original scale in this table but were z-score normalized before entering in the mixed model. ^2^Physiological stress was measured using participants’ heart rate and is the average BPM (beats per minute) during VR instruction after controlling for baseline heart rate. *p < .05; **p < .01; ***p < .001.

### Effects on physiological stress

First, we compared the goodness of fit between models. The change in log-likelihood was not significant when comparing M2 to M1 (∆χ^2^(2) = 4.06, *p* = .19), and M3 to M2 (∆χ^2^(2) = 1.86, *p* = .39), indicating that the goodness of fit did not significantly improve with the inclusion of the covariate of gender ($\widehat{\upbeta }$= 0.15, *p* = .59), nor the interaction terms of neuroticism/self-efficacy/cognitive (reasoning) abilities × complexity ($\widehat{\upbeta }$ = − 0.35/ − 0.24/3.50, *p* = .26/.47/.36). Thus, we chose to use M1 as the final model.

**Figure 3:**
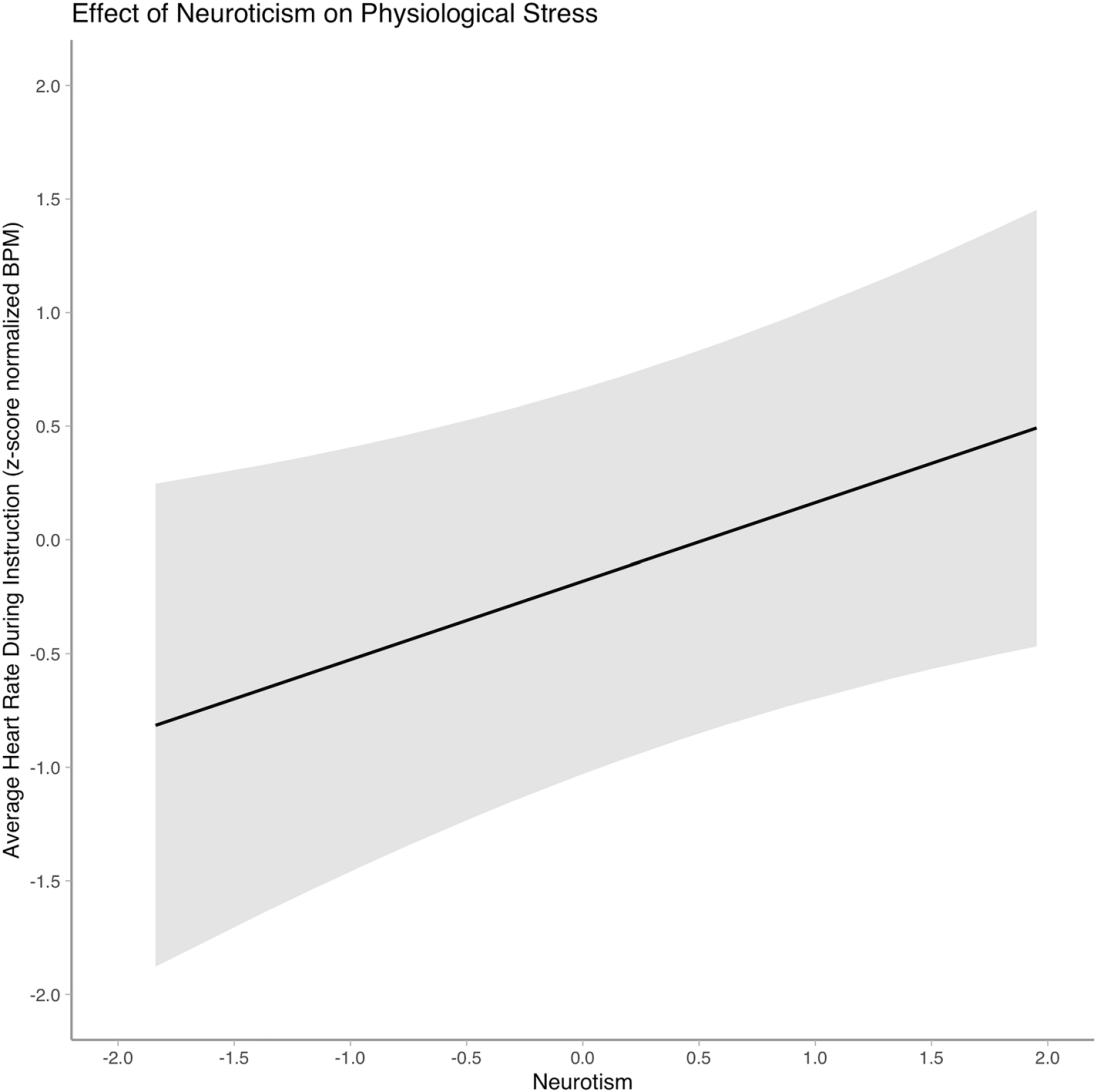
Effect of neuroticism on physiological stress (operationalized by heart rate).

Next, we examined participants’ physiological stress during the teaching task in VR. Participants’ HR during this task was on average 16.90 BPM higher than the baseline HR (Table 1). The effects of neuroticism, classroom management self-efficacy, cognitive (reasoning) abilities, and complexity were examined with M1. As shown in the model summary (Table 2, also see Supplementary Table S2 for the full model summary), neuroticism had a significant and positive effect on physiological stress ($\widehat{\upbeta }$ = 0.40, *p* = .01) with a large effect size (*d* = 0.76). This effect is visually represented in Fig. 3. We did not find statistically significant evidence regarding the main effects of classroom management self-efficacy ($\widehat{\upbeta }$ = − 0.41, *p* = .50), cognitive (reasoning) abilities ($\widehat{\upbeta }$ = − 0.06, *p* = .79), and complexity ($\widehat{\upbeta }$ = 0.26, *p* = .42).

**Table 2:** LMM model summary of Model 1.

Terms	^1^Physiological stress			^2^Psychological stress		
	$\widehat{\upbeta }$	*SE*($\widehat{\upbeta }$)	*t*	$\widehat{\upbeta }$	*SE*($\widehat{\upbeta }$)	*t*
Intercept	− 0.43	0.50	− 0.86	− 0.10	0.18	− 0.51
Complexity (large–small)	0.26	0.31	0.83	− 0.13	0.28	− 0.47
Neuroticism	0.40	0.15	2.59**	0.29	0.14	2.13*
Self-efficacy in classroom management	− 0.41	0.43	− 0.95	− 0.28	0.18	− 1.62
Cogn. abilities	− 0.06	0.19	− 0.32	0.002	0.14	0.01

*p < .05. **p < .01, ***p < .001. ^1^*n* = 47, AIC = 150.05, BIC = 178.49; ^2^*n* = 55, AIC = 169.89, BIC = 202.47. Cogn. abilities = Cognitive (reasoning) abilities. Physiological stress was operationalized by heart rate.

### Effects on psychological stress

Similarly, for psychological stress, the goodness of fit did not different significantly between M2 and M1 (∆χ^2^(2) = 1.77, *p* = .41), as well as between M3 and M2 (∆χ^2^(2) = 2.15, *p* = .29). The effect of the covariate of gender ($\widehat{\upbeta }$ = 0.36, *p* = .16), and the interaction terms neuroticism/self-efficacy/cognitive (reasoning) abilities × complexity ($\widehat{\upbeta }$ = 0.24/0.72/ − 0.14, *p* = .42/.08/.66) were not significant in relation to psychological stress. Therefore, we chose M1 as the final model to examine the fixed effects of neuroticism, classroom management self-efficacy, cognitive (reasoning) abilities, and complexity on psychological stress.

**Figure 4:**
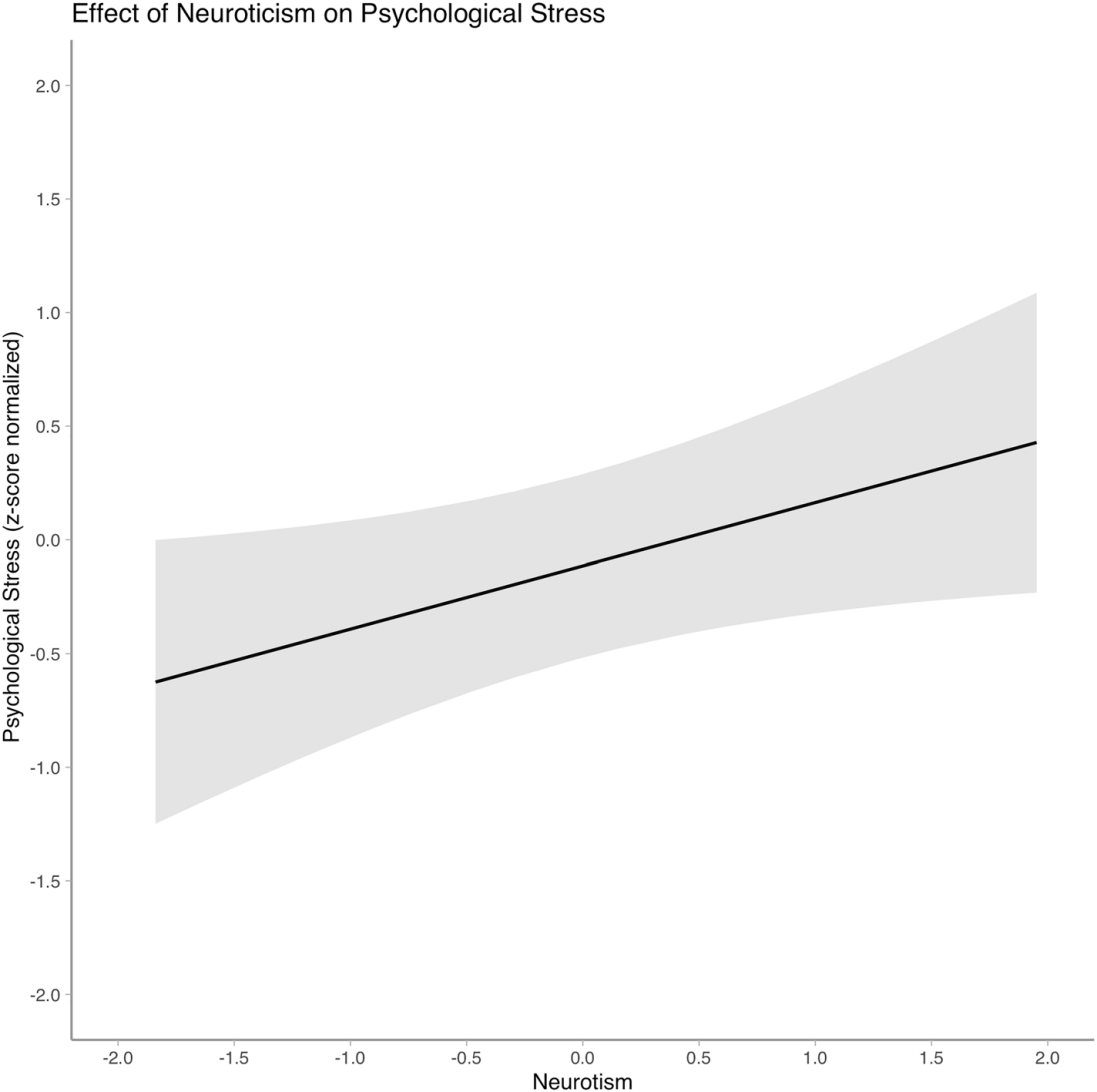
Effect of neuroticism on psychological stress.

As shown in Table 2 (also see Supplementary Table S3 for the full model summary), similar to physiological stress, neuroticism had a significant and positive effect on psychological stress ($\widehat{\upbeta }$ = 0.29, *p* = .04) with a medium to large effect size (*d* = 0.61). Figure 4 portrays this effect visually. The main effects of classroom management self-efficacy ($\widehat{\upbeta }$ = − 0.28, *p* = .16), cognitive (reasoning) abilities ($\widehat{\upbeta }$ = 0.002, *p* = .99), and of complexity ($\widehat{\upbeta }$ = − 0.13, *p* = .65) were not statistically significant.

## Discussion

Capitalizing on a VR classroom environment, this study aimed to investigate effects of pre-service teachers’ individual characteristics in terms of neuroticism, classroom management self-efficacy, and cognitive (reasoning) abilities on psychological and physiological stress responses. The results showed that neuroticism positively predicted psychological and physiological stress, whereas cognitive (reasoning) abilities and classroom management self-efficacy were not significantly related to either form of stress. We draw four major conclusions from our findings.

First, the results underscore the importance of neuroticism in research on stress^16^. As hypothesized, higher levels of neuroticism made pre-service teachers more vulnerable to experiencing stress in a fully standardized VR experimental environment. Educational psychologists are sometimes reluctant to include Big Five personality traits such as neuroticism in their research^77^. This likely stems from the misconception that Big Five personality traits are, unlike motivational constructs, stable, and therefore not a useful target for interventions. However, mounting evidence from personality psychology indicates that Big Five personality dimensions change over the lifespan^78^, are malleable, and can be altered via interventions, if people are motivated to change specific personality aspects^18,19^.

Our findings hold implications for research as they point towards the promise of focusing on neuroticism in research on stress in challenging teaching situations. The findings also have important implications for educational practice, for example, for teacher education programs and interventions for practicing teachers aiming to counteract neuroticism. People do, in general, want to become less neurotic. To illustrate, of all participants who signed up for a Big Five personality change intervention, most participants wanted to decrease in neuroticism (26.7%)^19^. Personality change interventions, which can be delivered digitally, could thus be used to support pre-service teachers and teachers to achieve their personal personality-change goals, such as reducing neuroticism levels^19^. Big Five personality change interventions typically include microinterventions (in terms of specific tools and techniques) that assist people in modifying or changing behaviors and experiences, and to maintain the change process^18,19^. In addition, mindfulness interventions have been shown to decrease neuroticism^79^, and could be more systematically integrated into teacher education programs and implemented in schools.

Second, there are theoretically sound reasons to assume that cognitive abilities should matter for how individuals, including teachers, deal with challenging situations^37,38^, which could affect their stress responses in such situations. Hence, we had hypothesized that higher cognitive (reasoning) abilities should predict lower levels of stress. However, according to our findings, pre-service teachers’ cognitive (reasoning) abilities did not play a relevant role for their psychological or physiological stress responses. Nonetheless, this finding adds to the scarce evidence base on teacher cognitive abilities and their correlates^37,80^. Future VR research on stress following up on our study could focus on potential links between teachers’ cognitive abilities and specific tasks within the VR environment that are even more “cognitively loaded” than the teaching task employed in our study (e.g., diagnostic decisions^38^).

Third, self-efficacy beliefs, and especially teaching-specific self-efficacy beliefs, have been portrayed as one of the most relevant teacher characteristics (e.g., regarding links to teachers’ occupational well-being, instructional approaches, and relations to student outcomes^81–83^) and have been conceptualized as personal resources that can counteract stress^23,83^. In our study, we had thus hypothesized that self-efficacy should be negatively related to stress. However, we did not obtain a significant effect of classroom management self-efficacy on stress experienced in the VR session. The reasons underlying this finding are hard to establish^84^. For instance, it could be that some of those pre-service teachers with higher classroom management self-efficacy also had higher expectations regarding their performance in the challenging teaching situation, which may have even caused stress. It may also be that classroom management self-efficacy does not exert an immediate effect within a stressful situation, which could have been captured in our study. Instead, effects of classroom management self-efficacy on stress responses may take longer to unfold and may to a larger extent depend on the broader social context^82^. More research systematically contrasting effects of self-efficacy on stress in “real-life” longitudinal studies^14^ and in VR environments is needed to gain clarity. Nonetheless, jointly, the non-significant effect for classroom management self-efficacy and the significant effect for neuroticism indicate that general behavioral tendencies (neuroticism) and not teaching-related characteristics (classroom management self-efficacy) seem more relevant to stress, even in a VR situation with a clear connection to classroom management (i.e., dealing with disruptive students). Against the background that research on teachers often strongly favors teaching-specific features, this is an important insight, which was made possible by the inter-theoretical integrative approach of our study focusing on individual characteristics from different research traditions. It is for future research to replicate our findings and to figure out whether neuroticism (and not teaching-specific self-efficacy beliefs) consistently predicts stress in other teaching situations.

Fourth, it should be mentioned that being assigned to one of the two complexity conditions (high versus low levels of disruptions) did not significantly predict stress. Further, we did not obtain significant interactions between individual characteristics and complexity levels. These findings speak to the fact that the investigated characteristics affected stress responses that would be generalizable to instructional situations with different complexity levels. Overall, our results are more in line with unidirectional perspectives on stress^21^ than with bidirectional perspectives presuming interactions between the person and the environment^24^, but replications using the same as well as combinations of other stressors are clearly required. Additionally, the finding that psychological and physiological stress reactions were low in correlation (see Table 1) but shared similar relationships with preservice teachers’ characteristics corresponds with earlier findings^85^ indicating that measuring stress with indicators of different modes is key for effects validation.

Fifth, our study underlines the value of using VR in psychological research in general and research on teacher characteristics and stress in particular. Prior survey-based research in “naturalistic” settings can be criticized for not being able to account for many confounding factors that may cause teacher stress, in addition to effects of individual characteristics. On the other hand, research in the lab often relies on artificial stress tasks that do not resemble the tasks that individuals encounter in their work life on a regular base and lack ecological validity. Hence, the reliance on VR in the present study helped to overcome significant limitations of prior research on (teacher) stress by balancing the teaching tasks with the precision afforded by experimental control^44^, thus combining the best from both worlds^43^. Moreover, it is interesting to note that in our study, neuroticism was significantly related to both psychological and physiological stress, whereas the meta-analysis by Luo et al.^16^ revealed significant links between neuroticism and psychological stress responses, but not physiological stress responses. Of course, replications of our work are needed; however, our findings may serve as an indication that VR environments that allow participants to experience work-like situations are particularly well suited to study effects of neuroticism on stress.

Several limitations and promising directions for future research should be noted. Our focus on the specific characteristics investigated in this study necessarily excluded other potentially relevant characteristics (e.g., other motivational constructs, such as causal attributions or interest, other Big Five personality traits^16^). In addition, even though the inclusion of both psychological and physiological stress responses is a strength of our study, considering a range of conventional stress measures (e.g., for physiological stress: heart rate variability indicators and salivary cortisol levels^8,86^) can provide an even more comprehensive understanding of the link between individual characteristics and stress. The dynamic nature of HR also calls for finer examinations of changes over time to capture time-lagged effects and the associations between time-varying contextual conditions with the cardiovascular data. One of the limitations of measuring HR in a room-scale VR simulation was the confounding influence of mental process and physical movement on HR measures. Future study may replicate the effect with more stationary setup to avoid potential movement artifacts. Further, we relied on measuring acute psychophysiological stress reactions in one short VR session, and it would have been desirable to investigate the exposure to multiple challenging VR situations and related changes in stress responses over time. Chronic stress reactions, which are long-term physiological and biochemical changes together with psychosomatic symptoms^87^ are also important to study in relation to individual characteristics. Relatedly, conceptual replications of our work using ecological momentary assessments to assess teachers’ daily hassles in naturalistic classroom settings^88^ could profitably complement VR-based research on stress. Also, the association between stress and teacher characteristics is not yet established for in-service teachers because the sample solely consisted of pre-service teachers who had not yet taught in actual classrooms. Further, some of our constructs were assessed with a few items only and had reliabilities slightly below .70. Hence, future research should use longer and more differentiated measures (e.g., distinct neuroticism facets). Another issue worth noting is that in our study classroom management self-efficacy and cognitive (reasoning) abilities had relatively lower standard deviations, which may have impacted the ability to detect significant effects. The cognitive abilities (reasoning) test was administered on the same date as the VR session. Even though it was not a high-stakes test situation (and no time limit was set for the test alone) and we do not think that the test caused a lot of stress, we cannot rule out that the test evoked stress in (some) participants. Hence, future VR research on cognitive abilities could administer the test at a separate day (or systematically vary time of administration to empirically examine whether working on a cognitive test prior to the VR session significantly affects stress). Finally, future work may want to adopt a within-person design in which participants complete both levels of complexity. However, in our study, we opted for a between-person design to reduce the burden for participants due to limited space and time resource, and because the issue relating to high versus low levels of complexity was not the main interest of our study. In our study, we were mainly interested in the effects of teacher characteristics on physiological and psychological stress.

To conclude, VR opens unprecedented opportunities for psychological research and the study of teaching and learning processes^89,90^. Our study adds to this emerging line of research by demonstrating that pre-service teachers’ neuroticism predicted psychological and physiological stress in a VR classroom. Classroom management self-efficacy and cognitive (reasoning) abilities were not significantly related to stress. Our results provide a basis for future studies to further exploit the potential of VR in order to examine characteristics relevant to teachers’ stress responses.

### Supplementary Information

Supplementary Tables.

## Data Availability

The anonymized dataset and all analysis files are available on the Open Science Framework (OSF, https://osf.io/vkbwx/). The source code of our virtual reality classroom (https://gitup.uni-potsdam.de/mm_vr/vr-klassenzimmer) is shared under GNU Affero General Public License.
