# Supplementary material for: Revisiting effects of teacher characteristics on physiological and psychological stress: a virtual reality study
Source: Sci Rep. 2023 Dec 14;13:22224. doi: 10.1038/s41598-023-49508-0 (PMC10721614; doi:10.1038/s41598-023-49508-0)
Supplement: Supplementary file 1 — Supplementary Tables. [file 41598_2023_49508_MOESM1_ESM.pdf]

Supplementary information

Revisiting Effects of Teacher Characteristics on Physiological and Psychological Stress: A Virtual Reality Study

written by Lisa Bardach, Yizhen Huang, Eric Richter, Robert M. Klassen, Thilo Kleickmann, and Dirk Richter

Time in seconds	Avatar location	Behavior	Category
330	02L	Play with a pen	off-task
345	02L	Write on the notebook	on-task
350	11R	Chat with the neighbor	off-task
365	11L	Write on the notebook	on-task
365	13R	Eat an apple	off-task
380	03R	Chat with the neighbor	off-task
380	14R	Stare outside the window	off-task
380	11L	Stare outside the window	off-task
400	01R	Hit the neighbor	off-task
420	05L	Throw paper balls	off-task
440	10R	Raise the arm	on-task
442	10R	Ask a question	on-task
470	12L	Eat an apple	off-task
480	11R	Chat with the neighbor	off-task
495	11R	Write on the notebook	on-task
500	02L	Play with a pen	off-task
515	13L	Hit the neighbor	off-task
530	13L	Write on the notebook	on-task
525	03L	Chat with the neighbor	off-task
540	03L	Write on the notebook	on-task
540	10R	Raise the arm	on-task
542	10R	Ask a question	on-task
560	07R	Stare outside the window	off-task

575	10R	Stare outside the window	off-task
575	11L	Stare outside the window	off-task
580	15R	Eat an apple	off-task
580	12L	Eat an apple	off-task
600	05L	Throw paper balls	off-task

Table S1. Behavior script of student avatars. This script initiates 330 seconds after the VR classroom starts to allow time for audio instruction. Students' behaviors were initiated independently of the teachers' actions. The order and category of student behaviors are randomized. The default behavior is "idle": agents would sit naturally in different neutral postures and move their eyes or bodies to follow the users around. After performing either on- or off-task behaviors (automatic termination after 15 seconds), agents would revert to the idle state.

	Fixed Effects			
	$\hat{\beta}$	*SE*($\hat{\beta}$)	95% CI	*t*
Intercept	−0.43	0.50	−1.40—0.55	–0.86
Complexity (large–small)	0.26	0.31	−0.35—0.87	0.83
Neuroticism	0.40	0.15	0.10—0.69	2.59**
Self-efficacy in classroom management	−0.41	0.43	−1.24—0.43	–0.95
Cognitive (reasoning) abilities	−0.06	0.19	−0.44—0.31	–0.32
	Random Effects			
			Variance	*SD*
Semester (Intercept)			0.14	0.38
Self-efficacy | Semester			0.07	0.26
VR Experience (Intercept)			0.40	0.64
Self-efficacy | VR Experience			0.29	0.53
	Model Fit			
R^2				Marginal
				0.19

Table S2. Full model summary of Model 1 for physiological stress. *n* = 47. **p* < .05. ***p* < .01, ****p* < .001. Marginal R^2 is a measure of the proportion of variance explained by the fixed effects[66]. Table was adapted from Meteyard and Davies[67]. Model specification: Physiological stress response ~ 1 + complexity + neuroticism + self-efficacy + cognitive (reasoning) abilities + (1 + self-efficacy | semester) + (1 + self-efficacy | VR experience). Physiological stress was operationalized by the average BPM during the VR teaching task after controlling for baseline HR.

	Fixed Effects			
	$\hat{\beta}$	$SE(\hat{\beta})$	95% CI	t
Intercept	−0.41	0.50	−1.39—0.57	−0.80
Complexity (large–small)	0.23	0.32	−0.40—0.87	0.72
Neuroticism	0.39	0.16	0.08—0.70	2.49*
Self-efficacy in classroom management	−0.39	0.42	−1.21—0.42	−0.95
Cognitive (reasoning) abilities	−0.07	0.21	−0.47—0.34	−0.32
Gender	0.15	0.28	−0.40—0.70	0.55
	Random Effects			
			Variance	SD
Semester (Intercept)			0.15	0.38
Self-efficacy | Semester			0.06	0.25
VR Experience (Intercept)			0.40	0.63
Self-efficacy | VR Experience			0.26	0.51
	Model Fit			
R^2				Marginal
				0.18

Table S3. Full model summary of Model 2 for physiological stress. $n = 47$. *$p < .05$. **$p < .01$, ***$p < .001$. Model specification: Physiological stress response ~ 1 + complexity + neuroticism + self-efficacy + cognitive (reasoning) abilities + gender + (1 + self-efficacy | semester) + (1 + self-efficacy | VR experience).

	Fixed Effects			
	$\hat{\beta}$	$SE(\hat{\beta})$	95% CI	*t*
Intercept	−0.43	0.31	−1.04—0.18	−1.39
Complexity (large–small)	−0.12	0.32	−0.73—0.50	−0.37
Neuroticism	0.39	0.16	0.08—0.70	2.52*
Self-efficacy in classroom management	−0.36	0.37	−1.08—0.37	−0.97
Cognitive (reasoning) abilities	−0.03	1.91	−3.77—3.70	−0.02
Gender	−0.01	0.28	−0.56—0.54	−0.04
Complexity * Neuroticism	−0.35	0.31	−0.96—0.25	−1.15
Complexity * Self-efficacy	−0.24	0.32	−0.85—0.38	−0.75
Complexity * Cognitive (reasoning) abilities	3.50	1.07	1.40—5.61	3.27
	Random Effects			
			Variance	*SD*
Semester (Intercept)			0.08	0.28
Self-efficacy | Semester			0.05	0.22
VR Experience (Intercept)			0.12	0.35
Self-efficacy | VR Experience			0.20	0.45
	Model Fit			
R^2				Marginal
				0.32

Table S4. Full model summary of Model 3 for physiological stress. $n = 47$. *$p < .05$. **$p < .01$, ***$p < .001$. Model specification: Physiological stress response ~ 1 + complexity + neuroticism + self-efficacy + cognitive (reasoning) abilities + gender + complexity:neuroticism + complexity:self-efficacy + complexity:cognitive (reasoning) abilities + (1 + self-efficacy | semester) + (1 + self-efficacy | VR experience).

	Fixed Effects			
	$\hat{\beta}$	$SE(\hat{\beta})$	95% CI	t
Intercept	−0.10	0.18	−0.46—0.27	−0.51
Complexity (large–small)	−0.13	0.28	−0.68—0.42	−0.47
Neuroticism	0.29	0.14	0.02—0.56	2.13*
Self-efficacy in classroom management	−0.28	0.18	−0.63—0.06	−1.62
Cognitive (reasoning) abilities	0.002	0.14	−0.26—0.27	0.01
	Random Effects			
			Variance	*SD*
Semester (Intercept)			0.06	0.05
Self-efficacy | Semester (Intercept)			0.12	0.34
VR Experience (Intercept)			0.02	0.15
Self-efficacy | VR Experience (Intercept)			0.06	0.25
	Model Fit			
R^2				Marginal
				0.17

Table S5. Full model summary of Model 1 for psychological stress. $n = 55$. *$p < .05$. **$p < .01$, ***$p < .001$. Marginal R^2 is a measure of the proportion of variance explained by the fixed effects[66]. Table was adapted from Meteyard and Davie[67]. Model specification: Psychological stress response ~ 1 + complexity + neuroticism + self-efficacy + cognitive (reasoning) abilities+ (1 + self-efficacy | semester) + (1 + self-efficacy | VR experience).

	Fixed Effects			
	$\hat{\beta}$	$SE(\hat{\beta})$	95% CI	t
Intercept	−0.11	0.24	−0.58—0.36	−0.47
Complexity (large–small)	−0.19	0.26	−0.71—0.32	−0.73
Neuroticism	0.28	0.13	0.02—0.54	2.10*
Self-efficacy in classroom management	−0.34	0.20	−0.73—0.05	−1.72
Cognitive (reasoning) abilities	0.02	0.14	−0.26—0.29	0.11
Gender	0.36	0.25	−0.13—0.85	1.44
	Random Effects			
			Variance	*SD*
Semester (Intercept)			0.05	0.22
Self-efficacy | Semester			0.22	0.47
VR Experience (Intercept)			0.08	0.27
Self-efficacy | VR Experience			0.70	0.84
	Model Fit			
R^2				Marginal
				0.20

Table S6. Full model summary of Model 2 for psychological stress. $n = 55$. *$p < .05$. **$p < .01$, ***$p < .001$. Model specification: Psychological stress response ~ 1 + complexity + neuroticism + self-efficacy + cognitive (reasoning) abilities + gender + (1 + self-efficacy | semester) + (1 + self-efficacy | VR experience).

	Fixed Effects			
	$\hat{\beta}$	$SE(\hat{\beta})$	95% CI	*t*
Intercept	−0.68	0.44	−1.54—0.18	−1.56
Complexity (large–small)	−0.09	0.26	−0.59—0.42	−0.33
Neuroticism	0.22	0.15	−0.07—0.51	2.41*
Self-efficacy in classroom management	−0.36	0.20	−0.76—0.03	−1.83
Cognitive (reasoning) abilities	0.02	0.15	−0.28—0.31	0.10
Gender	0.41	0.25	−0.08—0.90	1.66
Complexity * Neuroticism	0.24	0.30	−0.34—0.83	0.82
Complexity * Self-efficacy	0.72	0.37	−0.003—1.45	1.95
Complexity * Cognitive (reasoning) abilities	−0.14	0.31	−0.73—0.46	−0.45
	Random Effects			
			Variance	*SD*
Semester (Intercept)			0.02	0.15
Self-efficacy | Semester			0.19	0.44
VR Experience (Intercept)			0.08	0.29
Self-efficacy | VR Experience			0.10	0.24
	Model Fit			
R^2				Marginal
				0.25

Table S7. Full model summary of Model 3 for psychological stress. $n = 55$. *$p < .05$. **$p < .01$, ***$p < .001$. Model specification: Psychological stress response ~ 1 + complexity + neuroticism + self-efficacy + cognitive (reasoning) abilities + gender + complexity:neuroticism + complexity:self-efficacy + complexity:cognitive (reasoning) abilities + (1 + self-efficacy | semester) + (1 + self-efficacy | VR experience).

Construct	M (SD)/%		Range	
	Low Complexity	High Complexity	Low Complexity	High Complexity
Gender	42.31% cis female	56.67% cis female		
Semester	4.40 (3.02)	5.10 (4.20)	1–12	1–21
VR Experience	1.20 (0.41)	1.40 (0.56)	1–2	1–3
Neuroticism	3.22 (0.74)	2.69 (0.74)	2.00–4.67	1.33–4.00
Self-efficacy in Classroom Management	3.74 (0.49)	3.84 (0.73)	1.25–5.50	1.00–6.00
Cognitive (reasoning) abilities	5.31 (2.21)	6.52 (2.44)	1–10	1–10
Psychological Stress	2.70 (0.60)	4.17*** (1.33)	1.50–4.00	1.75–6.50
Physiological Stress	16.77 (12.63)	17.00* (15.60)	−5.22–38.33	−10.72–62.40

Table S8. Descriptive of included variables by levels of complexity. Cells are left blank if not applicable. Semester 1 (N_1 = 26) used low complexity, semester 2 (N_2 = 30) used high complexity. *$p < .05$. **$p < .01$, ***$p < .001$ when comparing high to low complexity. Multiple comparisons were corrected with Games-Howell method. Physiological stress was operationalized by heart rate.
